# First Record of *Calliephialtes sittenfeldae* Associated with the Tephritid Fruit Fly *Anastrepha spatulata* in Mexico

**DOI:** 10.1673/031.012.3401

**Published:** 2012-03-09

**Authors:** Maurilio López-Ortega, Andrey I. Khalaim

**Affiliations:** ^1^ lnstituto de Biotecnología y Ecología Aplicada (INBIOTECA), Universidad Veracruzana, Xalapa, Veracruz 91001, Mexico; ^2^ División de Estudios de Postgrado e Investigación, UAM Agronomía y Ciencias, Universidad Autónoma de Tamaulipas, Cd. Victoria 87149, México. Zoological Institute, Russian Academy of Sciences, Universitetskaya nab. 1, St. Petersburg 199034, Russia

**Keywords:** field observations, host, immobilized fly larva, Olacaceae, parasitoid, *Schoepfia schreberi*

## Abstract

This paper reports for the first time an ichneumonid parasitoid *Calliephialtes sittenfeldae* Gauld Ugalde-Gómez et Hanson (Hymenoptera: Ichneumonidae) associated with a dipteran host *Anastrepha spatulata* Stone (Diptera: Tephritidae), recovered from fruit of *Schoepfia schreberi* (Santalales: Olacaceae) in central Veracruz, Mexico. Large numbers of this parasitoid were collected and reared from its fruit fly host in three localities of Veracruz, Mexico. Some observations of its biology are also reported. This is a first record of *C. sittenfeldae* from Mexico, and the first record of this parasitoid species, its insect host, and the host plant. The male of this species is described and illustrated for the first time.

## Introduction

*Calliephialtes* Ashmead (Hymenoptera: Ichneumonidae) is a moderately large ichneumonid genus comprising about 22 species, and is confined to the New World (Gauld et al. 1998; Yu et al. 2005). Most species of this genus are found in the tropics and subtropics, although four species are known from USA and Canada (Townes and Townes 1960). Twelve species have been described or recorded from Costa Rica (Central America) (Gauld 1991; Gauld et al. 1998), and seven species from South America (Yu et al. 2005).

*Calliephialtes* is morphologically similar to the genera *Scambus* Hartig and *Anastelgis* Townes (Gauld 1991). It can be distinguished from *Scambus* by the absence of epomia, and cylindrical long ovipositor (3.6–5.6 times as long as hind tibia) with apex flexible downwards, with a scabrous area laterally, and from *Anastelgis* by the metasomal tergite 2 without oblique grooves anterolaterally, propodeum without lateromedian longitudinal carinae anteriorly, apex of ovipositor with a scabrous area laterally and without row of minute denticles on the upper valve, and the ovipositor sheath entirely black (all North and Central American species of *Anastelgis* have a black sheath with white apex).

Mexican fauna of *Calliephialtes* comprises at least six species. Two species, *C. coxatus* and *C. thurberiae*, were reported to occur in Mexico by Ruíz-Cancino et al. (2002). However, according to Gauld (1991), the first species is known from two specimens from Costa Rica only. Other material is actually *Scambus flavipes*, which was mistakenly considered as a synonym of *C. coxatus.* Thus, only one species, *C. thurberiae*, was known from Mexico until now. The widely distributed Caribbean species *C. ferrugineus* was recorded from the USA (Florida), Puerto Rico, Cuba, and Costa Rica (Gauld 1991; Yu et al. 2005), and is expected to occur in Mexico. In this paper we record *C. sittenfeldae* from Veracruz (Mexico), and its male is described for the first time.

All species of *Calliephialtes* are idiobiont ectoparasitoids of immature stages of various Lepidoptera and Coleoptera that live in galls, nuts, small fruit, mines, cases, and similar conditions (Brenner et al. 2002; Oboyski et al. 2004). One species, *C. grapholithae*, however, has been recorded as a facultative hyperparasitoid of a primary braconid parasitoid (Gauld 1991).

Tephritid parasitoids were studied in Chiapas, Mexico, by Aluja et al. (1990), but in spite of the large material, of more than 1300 reared parasitoids, no ichneumonid wasp was recorded. Until now most parasitoid species recorded for fruit flies belonged to the families Braconidae, Figitidae, Eulophidae, Diapriidae, Chalcididae, Eurytomidae, and Pteromalidae (Wharton 1998; Sivinski et al. 2000; Ovruski et al. 2004; Gates et al. 2008). Ichneumonidae quite rarely parasitize dipterous hosts, and few ichneumonid subfamilies are trophically connected with flies. Two ichneumonid subfamilies, Diplazontinae and Orthocentrinae, are specialized on dipterous hosts of Syrphidae and Mycetophilidae, and most other host records from Diptera belong to Cryptinae and Pimplinae (Hoffmeister 1992; Hagley et al. 1993). The present study reports, for the first time, a *Calliephialtes* species associated with Diptera, representing one of the few detailed records of an ichneumonid parasitoid on Tephritidae. *Calliephialtes sittenfeldae* is reported as a common parasitoid of the fruit fly *Anastrepha spatulata* Stone (Diptera: Tephritidae) infesting fruit of *Schoepfia schreberi* J.F. Gmel. (Santalales: Olacaceae) in Veracruz, Mexico, and details of the hostparasitoid relationship are given. Previously, from this fruit fly host, only two Braconidae parasitoids had been reported (Aluja et al. 2000). Although several species of *Anastrepha* in the same study region are parasitized by native and exotic hymenopterous larval-pupal parasitoid species (Lopez et al. 1999), most of them have been also found parasitizing diverse *Anastrepha* species, in others regions of Mexico (Aluja et al. 2003). Some parasitoid species exhibit a wide distribution and have been recovered from several species of *Anastrepha*, *Rhagoletotrypeta*, and *Ceratitis capitata.* In Mexico and Florida, they are used as tephritid biological control agents (Ovruski et al. 2000).

## Materials and Methods

Material was collected and field observations were made during 2006–2008 in the following three localities of Veracruz, Mexico:

1. “Tejeria”, 19° 21′ N, 96° 54′ W, altitude 924 m, municipality of Teocelo, represented by fragmented oak forest, intermixed with coffee plantations, and fruit-tree (e.g., orange, guava) orchards.
2. The conservation area named “Osto”, 19° 18′ N, 96° 50′ W, altitude 838 m, near the municipality of Tlaltetela. It is an area of fragmented oak forests mixed with dry forest, pasture grasslands, and sugar cane plantations.
3. “La Camelia”, 20° 84′ N, 101° 83′ W, altitude 237 m, municipality of Alamo. This locality is dominated by oak forest with tropical medium forest Subperennifolia (Puig 1991) surrounded by extensive citrus plantations.

The host *Schoepfia schreberi* (Santalales: Olacaceae) is a shrub that grows in low deciduous forests; dry oak forest and secondary vegetation. It is distributed from Venezuela throughout Central America and Mexico to Florida, and is also found in some Caribbean islands. It flowers from September to March, produces berries with a diameter of 5–12 mm, green in color when immature and red-dark when ripe. The seed is surrounded by a red aril (Sanchez 1996).

Field observations on *S. schreberi* trees (N = 4; 2.31 ± 0.80 m tall) were made systematically in Tejeria and Osto, from the 12 February through 26 of February in 2006 and 2007. Temperature and relative humidity were taken every 30 min with HOBO H8 Pro Series data loggers (Onset Computer Corporation, www.onsetcomp. com). Sixteen fruits of *S. schreberi* on which female parasitoids were observed ovipositing were taken to the laboratory. Half of the fruits (8) were dissected. Different stages of the life cycle were observed with a microscope; one fruit was dissected every three days, photographing each fruit with larvae using a Canon EOS Digital Rebel XTi digital camera (www.canon.com) and a MP-E 65mm Macro Photo lens (Canon). The other eight fruits were kept in No. 8530B sorting trays (Bioquip Products, Inc., www.bioquip.com) until the emergence of adults.

In Alamo, fruit of *S.* *schreberi* were collected on 3 March and 15 March. A random sample of 950 fruits was taken from 32 trees, and transported to a laboratory in Xalapa, Veracruz. Individual fruits were weighed (n = 200) and placed in sorting trays, then incubated at 17–21 °C until insect-parasitoids emergence.

22 males and females of the parasitoid *C. sittenfeldae* were reared from *A. spatulata* collected from *S. schreberi.* Catalogue of tephritid parasitoids and predators have been analyzed (Stibick 2004) and no host records of this group of parasitoids were found. Voucher specimens were placed in INBIOTECA (Universidad Veracruzana, Xalapa, Veracruz, Mexico), Universidad Autónoma de Tamaulipas (Cd. Victoria, Tamaulipas, Mexico), Zoological Institute of the Russian Academy of Sciences (St. Petersburg, Russia), TAMU, Texas A&M University (Texas, USA), and USNM National Museum of Natural History, Smithsonian Institution (Washington, D.C., USA).

## Results and Discussion

**Figure 1.**
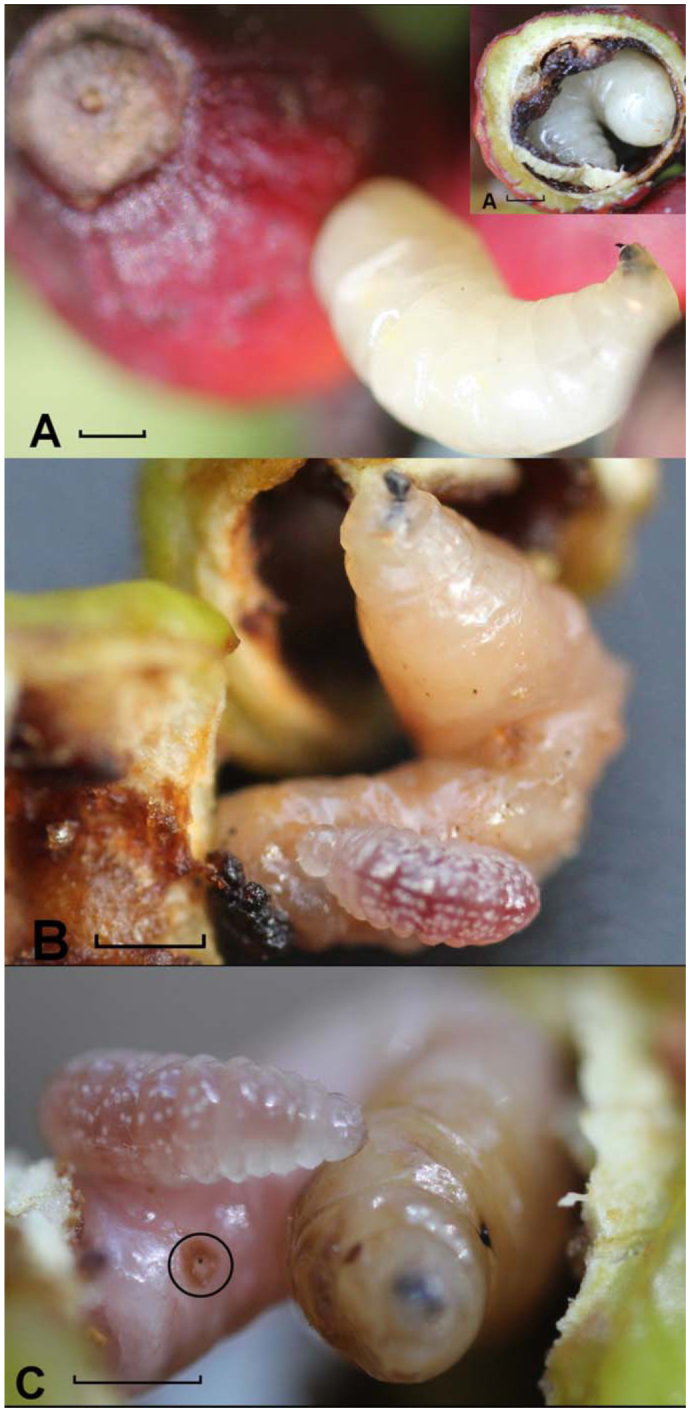
(A) Healthy larva of *Anastrepha spatulata* inside the host fruit and healthy larva fully mature leaving the host fruit. (B) Larva of *Calliephialtes sittenfeldae*, feeding on top of a larva of *Anastrepha spatulata.* (C) *Calliephialtes sittenfeldae* larva feeding on *Anastrepha spatulata.* Circle indicates the parasite bite. High quality figures are available online.

The fruiting season of *S. schreberi* takes place from early January to late March. Each fruit contained only one larva of *A. spatulata.* Females of *C. sittenfeldae* were observed from the 12 February to 26 February in 2006 and 2007. Host seeking activity was mostly recorded from 09:00 to 13:00 and from 15:00 to 17:00. The temperature ranged from 7.8 to 35.7 °C (average 18.6 °C) and relative humidity from 24.3 to 99.8% (Average 80.5). Female *C. sittenfeldae* were observed seeking for fruit fly hosts in ripe fruit only. This suggests that female parasitoids prefer to parasitize larvae of *A. spatulata* that have reached the third instar, because unripe fruit predominantly contain early instar larvae of the fly. Dissections of infested ripe fruit revealed that 100% of the larvae had depleted all the resource (the seed of the fruit) and all were fully mature, ready to leave the fruit and pupate (Figure 1A). Female parasitoids detected infested fruit, inserting the ovipositor six to nine times in the fruit, probably to immobilize the fly larva, and then layed the egg. Female parasitoids were not observed feeding on the fruit, but were seen making a circular cut on the skin of the fruit with using their mandibles. The function of this behavior is unknown, although we speculate that it might be to facilitate the departure of the offspring from the fruit. A single female parasitoid visited several branches in the same tree looking for infested fruit and oviposited only on the fruit that had a host. The whole immature life cycle of the parasitoid takes place inside the fruit of *S. schreberi*, and lasts approximately 24 ± 6 days.

Fruit taken at random from the trees where female parasitoids were observed ovipositing had an average fresh weight of 0.1885 ± 0.0618 mg, and their size was 6.7647 ± 0.4696 mm length, and 5.1070 ± 0.5736 mm width (n = 200). Uninfested fruit had an average fresh weight of 0.2272 ± 0.0572 mg, and their size was 7.4416 ± 0.6466 mm length, and 6.4273 ± 0.5722 mm in width (n = 60).

### Stages of development of *C. sittenfeldae*:

The egg of the parasitoid was laid close to the immobilized fly larva; as soon as it hatched the parasitoid larva started to feed on the fly larva, chewing a hole in the cuticle from which they sucked the juices of the host (Figure 1C). The parasitoid larvae develop very quickly, and are always very close to the immobile fly larvae (Figure 1B). All of the associations observed in dissected fruit were unmistakable because the distinctive posterior spiracles of the cyclorrhaphous dipteran were readily visible (Figure 1A). No record was taken on how many larval instars the parasitoid completed, but during its development it consumed the fly larva almost completely. After feeding, each parasitoid larva started to produce an oval cocoon. Thus, pupation takes place inside the fruit and not in the ground. Adults emerged after 24 days through a hole on the seed that they produce themselves. From the eight fruits collected that had been oviposited by the parasitoid in the field, three females and two males were obtained, and 10 males and 7 females were obtained from the other 950 fruit that were collected at random in Alamo. Other parasitoids of *Anastrepha* fruit flies have been obtained from pupae collected from the substrate after the larvae drop. Since the discovery of the biology of *C. sittenfeldae*, other parasitoids could be found in the fruits. This species could be using other species of the genus *Anastrepha* in other fruits. Other parasitoid species infest fruit flies with native and exotic hosts at different altitudes in Veracruz (Sivinski et al. 2000). Furthermore, it seems that *C. sittenfeldae* is a widely distributed species, as it was found on altitudes ranging from 237 m to 924 m in Veracruz. Some species of ichneumonid wasps were reared from tephritids, especially in Europe and North America; almost all these host records belong to idiobiotic puparium ectoparasitoids of Cryptinae, mostly the genera *Phygadeuon*, which attack hosts after pupation in the soil (e.g., Hoffmeister 1992; Hagley et al. 1993). According to Stibick (2004), *Pimpla pomorum* in Spain and *Isurgus* sp. in Africa also may be parasitoids of tephritid flies, but the first record is doubtful, and the second is incorrect. Thus, at present day there is a not reliable record of Pimplinae from a tephritid host.

**Figure 2.**
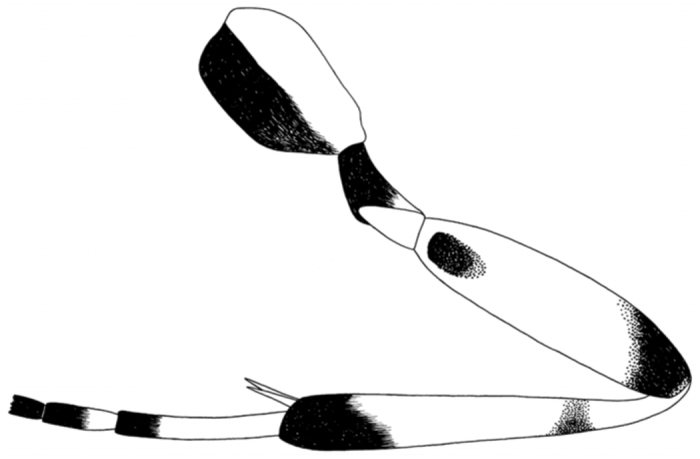
*Calliephialtes sittenfeldae* male. Hind leg without apical tarsomeres, lateral view. High quality figures are available online.

**Figure 3.**
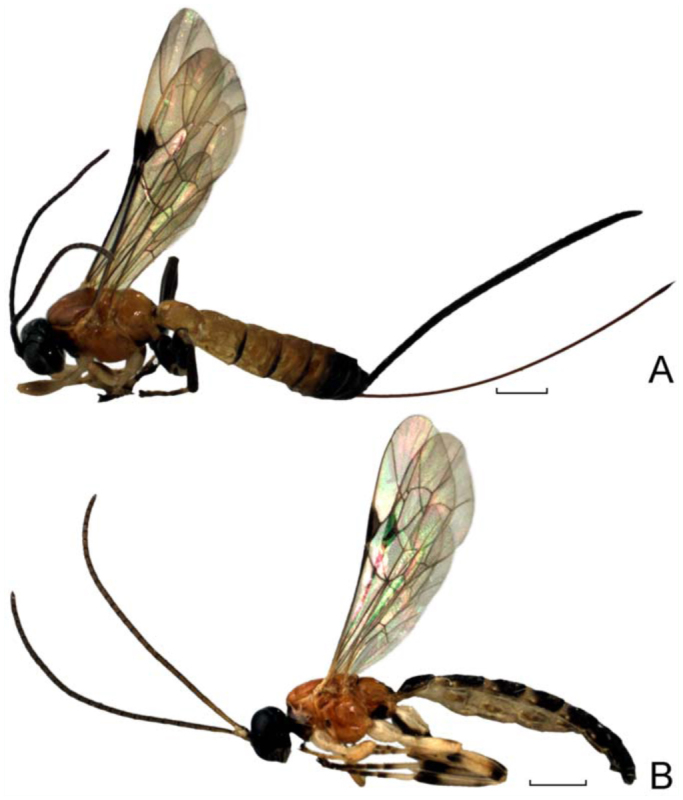
*Calliephialtes sittenfeldae*, complete body, lateral view. (A) female, (B) male. High quality figures are available online.

### Description of male of *C. sittenfeldae* (Figures 2, 3).

Forewing length 4.6–5.6 mm. Mandible longitudinally impressed, with upper tooth about as long as lower tooth. Malar space narrow, 0.12–0.18 times as long as basal width of mandible. Eye orbits, in frontal view, weakly, but distinctly convergent ventrally. Head, in dorsal view, with genae roundly narrowed behind eyes. Lateral ocellus separated from eye by 0.9–1.0 times its own maximum diameter. Occipital carina weak dorsally and laterally. Flagellum of antenna with 25–26 segments. Face shagreen and dull to almost smooth, rather densely pubescent. Frons and temple polished, sparsely pubescent.

Mesosoma polished. Pronotum unspecialized. Propodeum finely punctate dorsolaterally. Pleural carina present. Submetapleural carina represented by a crest anteriorly, posteriorly absent. Wings hyaline. Hind wing with nervellus intercepted somewhat above middle.

First tergite of metasoma 1.16–1.20 times as long as posteriorly broad, with lateromedian carinae more or less complete, converging towards centre of tergite, strong anterolaterally and sometimes evanescent posteriorly, and lateral longitudinal carina strong, reaching to posterior margin of tergite; first tergite smooth dorso-anteriorly, uneven laterally and dorso-posteriorly, with coarse punctures in posterior half. Second tergite distinctly elongate, almost entirely densely punctate, with posterior margin impunctate, more or less smooth between punctures. Tergite 3–7 similar through progressively more weakly punctate.

Head and antenna black. Scape and pedicel of antenna ventrally, and palpi whitish. Base of flagellum more or less brownish ventrally. Mesosoma predominantly reddish orange. Propleuron entirely blackish. Pronotum widely blackish in anterior part, reddish orange dorso-posteriorly. Tegula whitish. Pterostigma dark brown. Metapleuron blackish ventrally. Propodeum with anterior margin blackish, widely blackish in posterior part, or sometimes propodeum almost entirely blackish. Legs whitish. Foreleg usually with femur and tibia slightly infuscate dorsally, and tarsus more or less infuscate. Mid leg with femur infuscate dorsally, tibia infuscate dorsally and apically, tarsomeres 1–2 fuscous apically, tarsomeres 3–5 more or less entirely fuscous. Hind leg with coxa blackish ventrally, trochanter blackish with dorsoposterior whitish mark, femur with a pair of blackish subbasal marks on its inner and outer surfaces and blackish apically, tibia blackish subbasally and apically (Figure 1), tarsomeres 1–2 blackish apically, tarsomeres 3–5 more or less entirely blackish. Metasomal tergites fuscous, tergite 2 with thyridia pale, tergites 3– 5 with pale band anteriorly.

## Editor's note

Paper copies of this article will be deposited in the following libraries. Universitaetsbibliothek Johann Christian Senckenberg, Frankfurt Germany; National Museum of Natural History, Paris, France; Field Museum of Natural History, Chicago, Illinois USA; University of Wisconsin, Madison, USA; University of Arizona, Tucson, Arizona USA, Smithsonian Institution Libraries, Washington D.C. USA; The Linnean Society, London, England. The date of publication is given in ‘About the Journal’ on the JIS website.
